# Temporal order of evolution of DNA replication systems inferred by comparison of cellular and viral DNA polymerases

**DOI:** 10.1186/1745-6150-1-39

**Published:** 2006-12-18

**Authors:** Eugene V Koonin

**Affiliations:** 1 National Center for Biotechnology Information, National Library of Medicine, National Institutes of Health, Bethesda, MD 20894, USA

## Abstract

**Background:**

The core enzymes of the DNA replication systems show striking diversity among cellular life forms and more so among viruses. In particular, and counter-intuitively, given the central role of DNA in all cells and the mechanistic uniformity of replication, the core enzymes of the replication systems of bacteria and archaea (as well as eukaryotes) are unrelated or extremely distantly related. Viruses and plasmids, in addition, possess at least two unique DNA replication systems, namely, the protein-primed and rolling circle modalities of replication. This unexpected diversity makes the origin and evolution of DNA replication systems a particularly challenging and intriguing problem in evolutionary biology.

**Results:**

I propose a specific succession for the emergence of different DNA replication systems, drawing argument from the differences in their representation among viruses and other selfish replicating elements. In a striking pattern, the DNA replication systems of viruses infecting bacteria and eukaryotes are dominated by the archaeal-type B-family DNA polymerase (PolB) whereas the bacterial replicative DNA polymerase (PolC) is present only in a handful of bacteriophage genomes. There is no apparent mechanistic impediment to the involvement of the bacterial-type replication machinery in viral DNA replication. Therefore, I hypothesize that the observed, markedly unequal distribution of the replicative DNA polymerases among the known cellular and viral replication systems has a historical explanation. I propose that, among the two types of DNA replication machineries that are found in extant life forms, the archaeal-type, PolB-based system evolved first and had already given rise to a variety of diverse viruses and other selfish elements before the advent of the bacterial, PolC-based machinery. Conceivably, at that stage of evolution, the niches for DNA-viral reproduction have been already filled with viruses replicating with the help of the archaeal system, and viruses with the bacterial system never took off. I further suggest that the two other systems of DNA replication, the rolling circle mechanism and the protein-primed mechanism, which are represented in diverse selfish elements, also evolved prior to the emergence of the bacterial replication system. This hypothesis is compatible with the distinct structural affinities of PolB, which has the palm-domain fold shared with reverse transcriptases and RNA-dependent RNA polymerases, and PolC that has a distinct, unrelated nucleotidyltransferase fold. I propose that PolB is a descendant of polymerases that were involved in the replication of genetic elements in the RNA-protein world, prior to the emergence of DNA replication. By contrast, PolC might have evolved from an ancient non-templated polymerase, e.g., polyA polymerase. The proposed temporal succession of the evolving DNA replication systems does not depend on the specific scenario adopted for the evolution of cells and viruses, i.e., whether viruses are derived from cells or virus-like elements are thought to originate from a primordial gene pool. However, arguments are presented in favor of the latter scenario as the most parsimonious explanation of the evolution of DNA replication systems.

**Conclusion:**

Comparative analysis of the diversity of genomic strategies and organizations of viruses and cellular life forms has the potential to open windows into the deep past of life's evolution, especially, with the regard to the origin of genome replication systems. When complemented with information on the evolution of the relevant protein folds, this comparative approach can yield credible scenarios for very early steps of evolution that otherwise appear to be out of reach.

**Reviewers:**

Eric Bapteste, Patrick Forterre, and Mark Ragan.

## Open peer review

This article was reviewed by Eric Bapteste, Patrick Forterre, and Mark Ragan.

For the full reviews, please go to the Reviewers' comments section.

## Background

DNA replication is central to the reproduction of all cellular life forms and many viruses. Indeed, inasmuch as accurate DNA replication is strictly required for the faithful transmission of the information stored in the genomes of all known cellular life forms, it can be legitimately viewed as the quintessential biological process, the crucial manifestation of the proverbial double helix. Furthermore, mechanistically, the DNA replication processes in all cells, indeed, appear to be very similar [1]. Thus, it came as an extraordinary surprise when comparative genomics ushered in the realization that the protein components of DNA replication systems are not at all universally conserved [2-5], in a sharp contrast to the core parts of the translation and transcription systems that are, indeed, shared by all cellular life [6,7]. Notably, this dramatic disparity of DNA replication systems has been predicted in the seminal early work of Woese and Fox in the context of their concept of the Last Universal Common Ancestor (LUCA) of modern cellular life forms as a primitive entity, the progenote [8].

The DNA replication systems of bacteria, on the one hand, and archaea and eukaryotes, on the other hand, are a peculiar mix of conserved and unrelated proteins. Notably, the central parts of the machinery, in particular, the polymerases that are responsible for DNA chain elongation, primer formation, and gap filling after primer removal, and replicative helicases, are either unrelated or distantly related and are thought to derive independently from proteins with other functions [5,9]. The conclusion that the main replicative polymerases and primases of archaea (and eukaryotes) and bacteria are unrelated was, originally, reached through exhaustive protein sequence analysis [5]. Subsequently, this conclusion received crucial support from the solution of the crystal structures of the bacterial and archaeal primases [10-13] and replicative polymerases [14-17]. The comparison of the respective structures unequivocally demonstrated that they, indeed, have unrelated folds. The distinction between the archaeal and bacterial DNA replication systems is additionally emphasized by the discovery of a unique DNA polymerase that is involved in replication in euryarchaea [18,19]. However, several ancillary components, such as the sliding clamp (the proliferating cell nuclear antigen, PCNA, and its homologs), the clamp loader ATPase, and RNAse H are represented by well-conserved orthologs in bacteria and archaea (eukaryotes) [5].

This unexpected divergence of cellular DNA replication systems is, in principle, compatible with at least three distinct evolutionary scenarios each of which takes as the focal point the nature of the replication system that is inferred for the LUCA [5,20]. These three views of LUCA's genome replication go as follows: i) LUCA had no DNA replication per se but instead had a retrovirus-like replication cycle, with segments of genomic RNA reverse-transcribed into a DNA provirus, which is transcribed back into RNA; the existence of a DNA stage would explain the conservation of some proteins involved in DNA replication [5], ii) LUCA had one of the two of the modern types of DNA replication systems, either (proto)archaeal or (proto)bacterial; subsequent non-orthologous gene displacement of the key components in one of the primary lines of descent, possibly, via a virus vector, resulted in the current dichotomy[4,21], and iii) LUCA had both DNA replication systems (with one, possibly, involved in repair), with subsequent differential loss of the central components in the respective common ancestors of archaea and bacteria [2,4,21].

The notion of a possible contribution of DNA-containing viruses to the evolution of the DNA replication systems of cellular life forms has been presented in a series of publications by Forterre [21-24]. Recently, this line of thought has been further developed in two more general treatises each of which emphasized the integral connection between the evolutionary histories of cells and selfish genetic elements. The first of these studies posited that LUCA was a cell with an RNA genome, and the transition to the modern-type DNA replication system occurred after the divergence of the three primary lines of descent of cellular life forms, the progenitors of bacteria, archaea, and eukaryotes [25]. The second study laid out the argument for a non-cellular, although complex and compartmentalized LUCA, envisaged as a stage of evolution at which the progenitors of the main lineages of extant viruses already coexisted with elements that gave rise to bacterial and archaeal genomes [26]. Here I employ comparative genomics of viruses and cellular life forms to address a specific aspect of the evolution of DNA replication systems, namely, the temporal order of their emergence, and discuss the conclusions in conjunction with the general views on the nature of LUCA.

## Results

### The hypothesis: inferring the temporal order of the origin of DNA replication systems from comparison of viral and cellular genomes

Viruses possess a remarkable collection of diverse genome replication and expression strategies, in a sharp opposition to the uniformity of the cellular genetic cycle [27,28]. Since the subject of this article is origin and evolution of DNA replication systems, I concern myself only with those viruses that possess DNA genomes; most, though not all, of these viruses encode their own core replication proteins. There are three basic types of viral DNA replication, one of which is, essentially, the same as the replication mode of cellular life forms (some interesting variations in viruses notwithstanding) whereas the remaining two – the protein-primed and the rolling circle replication (RCR) systems – seem to be unique to viruses and other selfish genetic elements (Table 1). A remarkable aspect of viral DNA replication that, to my knowledge, has never been interpreted in evolutionary terms, is that the vast majority of viruses with the "cell-like", RNA-primed, and terminal-protein-primed replication strategies encode the archaeal-type B-family DNA polymerase (hereinafter PolB) [29,30]. A series of exhaustive, iterative PSI-BLAST [31,32] searches of the viral subset of the non-redundant protein sequence database (NCBI, NIH, Bethesda) with bacterial PolC sequences as queries yielded only 8 bacteriophage PolC homologs (Table 1), in a sharp contrast to the thousands of viruses that possess PolB homologs (Table 1 and data not shown). The remaining viruses either have the A-family polymerase (hereinafter PolA) that performs, mostly, repair-related functions in bacteria (Table 1) or no DNA polymerase at all. No virus-encoded homologs of the unique euryarchaeal polymerase (hereinafter PolD) were identified. The other genes involved in viral DNA replication are a complex mix of homologs of bacterial and archaeal replicative proteins and virus-specific proteins [26,33,34] but the decidedly non-uniform distribution of polymerases is striking.

**Table 1:** Distribution of replicative DNA polymerases and distinct DNA replication systems among cellular life forms and viruses

	RNA-primed DNA replication				Protein-primed DNA replication	RCR system of ss/dsDNA replication
	bacterial-type//PolC	Bacterial/PolA	Archaeal-type (polB/D)		PolB	
					
			PolB	PolD		

Cellular life forms	Bacteria	Mitochondria	Archaea	Euryarchaea	none	none

Viruses and other selfish elements	8 bacteriophages: -Saccharomonospora phage PIS 136 (AAL66178) – unclassified bacteriophage -Mycobacteriophage PBI1 (YP_655259) -Siphoviridae -Mycobacteriophage Plot (YP_655445) – Siphoviridae -Mycobacteriophage Catera (YP_656181) – Siphoviridae -Mycobacterium phage Bxz1 (NP_818250)- Myoviridae -Mycobacteriophage Barnyard (NP_818618) – Siphoviridae – Bacteriophage SPBc2 (NP_046685) – Siphoviridae Clostridium botulinum phage C-St (YP_398491) – unclassified Caudovirales	T-odd and related bacterio-phages (Podoviridae)	Bacteriophages: myoviridae (e.g., T-even phages); some bacteriophages of the family Siphoviridae; Eukaryotic viruses: NCLDV^a^, herpesviridae, baculoviridae, some eukaryotic linear plasmids	none	Tectivirdae (e.g., phage PRD1), many bacteriophages of the family Siphoviridae (e.g., φ29); adenoviridae; linear plasmids from fungal and plant mitochondria	Bacteriophages: microviridae (e.g., φX174). Eukaryotic viruses with small ssDNA genomes: parvoviridae, nanoviridae, circoviridae, geminiviridae, numerous bacterial and archaeal plasmids

Viral taxonomy was the from the NCBI Taxonomy site [58] and is based on the 7^th ^report of the International Committee on Taxonomy of Viruses [59].

^a^NCLDV, nucleo-cytoplasmic large DNA viruses (poxviridae, asfraviridae, ascoviridae, iridoviridae, phycodnaviridae, mimivirus) [33].

**Figure 1:**
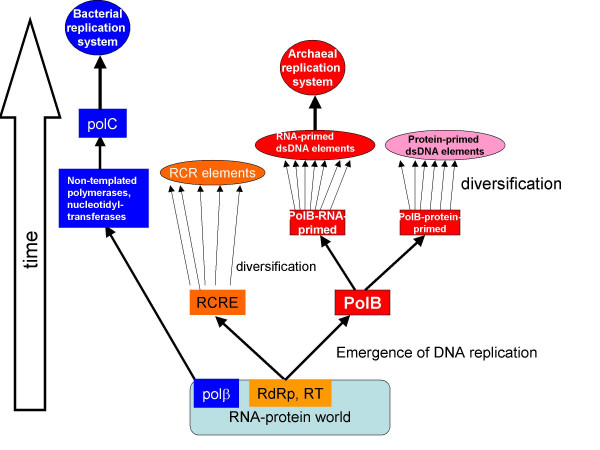
The inferred temporal order of evolution of DNA replication systems.

There is hardly any mechanistic impediment to the involvement of PolC in viral DNA replication as evidenced, in particular, by the presence of the gene for the PolC homolog in 8 phage genomes (Table 1). Additionally, a variety of bacteriophages, such as temperate phages of the family Siphoviridae, successfully recruit bacterial PolC for their replication but not the *polC *gene to their genomes. Furthermore, a direct comparison of the catalytic efficiencies of the three DNA polymerases of *E. coli*, PolA, PolB, and PolC, shows that PolC has a much greater turnover rate than the other two, i.e., is a substantially more efficient enzyme ([35,36]; and see Table 5-I in [1]). Thus, inasmuch as mechanistic causes for the dominance of PolB among viruses and the near absence of PolC are unlikely to exist, I propose a historical explanation (Fig. 1). I hypothesize that PolB is the most ancient replicative DNA polymerase and, accordingly, the archaeal-type DNA replication system centered around this polymerase was the first to have evolved among the two known cellular replication systems. Moreover, there was a time interval after the emergence of the PolB-centered, archaeal-type DNA replication system and before the advent of the bacterial, PolC-centered one, during which several lineages of selfish genetic elements with diverse life styles have emerged. In particular, the divergence between the RNA-primed and protein-primed branches of the PolB family of polymerases, each of which spans a broad range of viruses and other selfish elements [30,37], can be confidently assigned to this early stage of evolution. Perhaps, along with RCR elements, which also display remarkable diversity [38] and are likely to be of ancient origin, these viruses and virus-like entities have occupied the major existing biological niches and thus prevented any significant diversification of selfish elements carrying PolC. The presence of PolC in several bacteriophages (Table 1) might be the result of relatively late non-orthologous gene displacement, a phenomenon that seems to have occurred on several occasions during the evolution of DNA polymerases [30]. Indeed, the phage PolC sequences did not appear to be closely related to each other but instead showed the closest similarity to different bacterial polymerases (data not shown).

### Complications and caveats

Several compounding factors merit consideration in connection with this hypothesis. Firstly, and probably, most importantly, the current sampling of the "virosphere" is obviously incomplete. Only four major bacterial lineages (Proteobacteria, Cyanoabacteria, low-GC Gram-positive bacteria), two lineages of archaea (Sulfolobales and Halobacteria), and animals among the eukaryotes (as far as DNA viruses with large genomes are concerned) have been extensively sampled by viral genomics; there are only a few sequenced genomes of viruses infecting organisms outside these taxa. However, despite this limited sampling, the diversity of viruses with sequenced genomes is substantial by any criterion, be it replication strategy, genome size, gene repertoire, or virion structure. Therefore, it seems unlikely (although, certainly, not impossible) that sequencing of viruses from other lineages will radically change the distribution of DNA polymerases among viruses by revealing a dominant presence of PolC. Interestingly, in a recent study on viral metagenomics, a claim has been made that PolC is one of the dominant viral enzymes in three distant and diverse habitats [39]. However, examination of the lists of other enzymes that appeared to dominate these "viromes" indicates that these are uncharacteristic of viruses and, at lest in some case, unlikely to be present in a virus given their well-characterized functions (see the **Author Response **to Forterre below for additional details). Thus, these metagenomic results, mostly likely, reflect contamination of the analyzed viral samples with bacterial DNA and do not point to hidden diversity of viruses replicated by PolC appears unlikely.

The second complication for the present hypothesis is that many DNA viruses of archaea and bacteria encode no DNA polymerase of their own and employ the respective host enzymes. Among bacteriophages, the forms with and without virus-encoded replicative enzymes are interspersed within the same viral families which is best compatible with the latter having been derived by degenerative evolution. However, the case of the viruses of hyperthermophilic crenarchaea is truly mysterious. None of these viruses encode their own polymerase, and mostly, they do not possess any other viral hallmark genes either, with the exception of a single group that has the widespread icosahedral capsid protein [40,41]. The provenance of these viruses remains unclear: they might be ultimate derivatives of the virus world that have lost all its hallmarks (the more likely possibility in the context of the virus world concept [26]), or else, they might have evolved anew via assembly of genes derived from the host. Whichever of these scenarios turns out to be correct, these viruses do not possess PolC but, instead, are replicated by the host PolB. Thus, their unique gene repertoire might pose a challenge to the virus world concept but hardly undermines the present hypothesis.

The third problem is the relevance (or lack thereof) of the eukaryotic DNA viruses, which account for a good part of the overall viral diversity, and in particular, the preponderance of PolB in the replication systems (Table 1), for the problem of the ultimate origins of those systems. Indeed, origin of eukaryotes via the archaeal-bacterial symbiosis which, I believe, is, by far, the most likely scenario [42,43], implies that eukaryotic viruses are much younger than the viruses of archaea and bacteria. However, that does not automatically mean that the gene composition of eukaryotic viruses tell us nothing about the earliest stages of the evolution of genome replication. Indeed, considering the accumulating evidence that sampling of genes from bacterial and archaeal viruses was the primary route of origin of eukaryotic viruses [26], the gene repertoire of eukaryotic viruses would reflect the composition of the gene pool of archaeal and bacterial viruses at the time of eukaryogenesis, perhaps, ~2 billion years ago. Hence, the predominance of PolB in eukaryotic viruses suggests that the this was the primary viral DNA polymerase at that stage of evolution.

Finally, from the most general standpoint, the approach employed here is an extension of the traditional logic of the argument from diversity that is common, e.g., in phylogeography. Under this view, the area with the greatest diversity of representatives from a given taxon is considered to be the birthplace of the group (e.g., [44]). This is, essentially, a parsimony-type argument that might fail under special circumstances, such as a sweep of the entire habitat by a particularly fit form leading to the obliteration of the ancestral diversity and followed by a new diversification. Applied to the evolution of the replication systems, this would translate into the sweep of the virus world by PolB via extensive horizontal gene transfer (HGT), at a relatively late stage of evolution. It has been demonstrated that HGT is common in the evolution of DNA polymerases including the B family [30]. However, as discussed above, it is hard to think of a selective advantage of PolB that would trigger a massive sweep. The alternative possibility of a major bottleneck in the evolution of viruses followed by a non-selective takeover by PolB is not supported by any concrete evidence either. Thus, although it is impossible to formally rule out the possibility of a PolB sweep, this scenario appears unlikely.

### Support from the evolutionary relationships between DNA and RNA polymerases

The order of emergence of the replication systems proposed here seems to get support from the homologous relationships between DNA and RNA polymerases inferred from structural and sequence comparisons. The catalytic domain of PolB has the widespread palm-and-fingers fold [14,45] various modifications of which are also found in PolA [46] and in RNA-dependent RNA polymerases (RdRp) of RNA viruses and reverse transcriptases [47]. Notably, the key protein of rolling circle replication, the initiation endonuclease (RCRE), has a derived form of the same fold [48,49]. By contrast, the core domain of PolC [16,17] belongs to the unrelated fold of the polβ family that includes a variety of non-replicative nucleotidyltransferases, such as polyA polymerases [50]. The prevailing current scenario for the early evolution of life has DNA replication evolving from within a RNA-protein world where only RNA replication occurred, with reverse transcription being a likely intermediate stage of evolution [5,23,25,26]. Under this scenario, it appears most likely that PolB, PolA, and RCRE evolved from the ancient replicative enzymes (RdRp or, more likely, reverse transcriptase). In contrast, the ancestor of PolC, probably, originated as a non-specific, non-replicative polymerase, such as a polyA polymerase, and was recruited for the bacterial-type replication system at a later stage of evolution (Figure 1).

## Discussion and Conclusion

**Figure 2:**
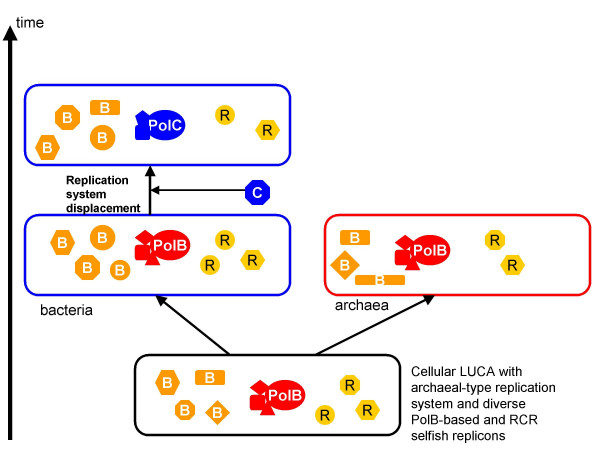
**Origin of DNA replication systems: the "archaeal LUCA" scenario**. Various small shapes denote viruses and other selfish replicons; B indicates elements encoding PolB, and R indicates elements encoding RCRE.

The hypothesis on the temporal order of the emergence of DNA replication systems proposed here is drawn directly from the data on the remarkably non-uniform distribution of DNA polymerases among viruses and virus-like elements and, accordingly, is not tightly linked to any specific model of the origin of cells and viruses. It is, nevertheless, interesting to consider how this hypothesis plays out in the context of two classes of such models. The first view which, conceivably, represents the orthodoxy, holds that the main classes of viruses emerged from already formed cells, probably, at early stages of evolution. Under this model, the present hypothesis implies that LUCA had the archaeal-type system of DNA replication, whereas the displacement of this ancestral system in bacteria, possibly, mediated by a virus [21,24], was a relatively late event (Figure 2).

**Figure 3:**
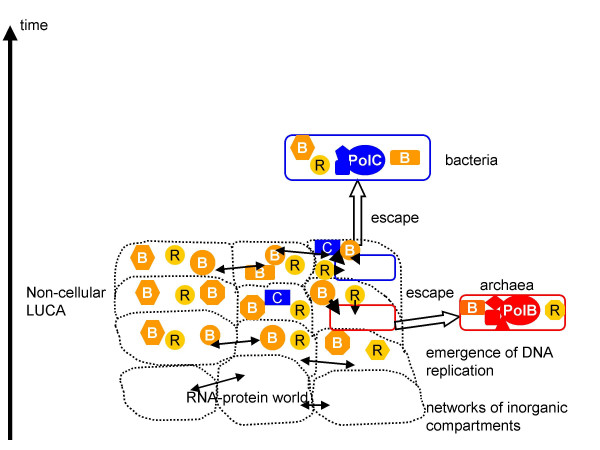
**Origin of DNA replication systems: the primordial gene pool scenario**. The schematic is based on models of pre-cellular evolution discussed in [21,38]. The walls of the inorganic compartments are shown by dotted lines to emphasize their porosity. Double-headed arrows denote inter-compartmental horizontal gene transfer. C denotes a hypothetical precursor of PolC, probably, a non-templated polymerase (see text). Other designations are as in Figure 2.

The alternative scenario [26] derives both virus-like elements and cells directly from a primordial gene pool (Figure 3). Under this view, LUCA did not have a cellular organization at all but instead consisted of a population of genetic elements that replicated and expressed proteins within networks of inorganic compartments[51,52]. This model stems from the lack of homology between the core components of the DNA replication systems and membrane biogenesis pathways in archaea and bacteria [5,53]. Accordingly, it is proposed that proto-archaeal and proto-bacterial cells escaped from these networks independently, following the evolution of the corresponding distinct versions of the membrane biogenesis machinery [51,52]. In conjunction with this model, the concept of the ancient virus world has been recently developed, according to which the major classes of viruses (more precisely, virus-like elements inasmuch as a pre-cellular stage of evolution is concerned) evolved already in the primordial gene pool, and distinct complements of viruses were captured by the escaping proto-archaeal and proto-bacterial cells [26]. Under this scenario, the present hypothesis implies that genetic elements encoding PolB as well as those with the RCR mode of replication have evolved considerable diversity prior to the emergence of cells. By contrast, the bacterial replication system was "invented" later and was recruited by a very limited range of bacteriophages, possibly, at much later stages of evolution. The evolutionary status of the PolA-based replication system, which is found in a limited range of bacteriophages (Table 1) and is centered around a polymerase that is involved in gap-filling during replication and in repair in all bacteria[1], is less clear. A progenitor of the PolA-replicated phages might have evolved already at an early, perhaps, pre-cellular stage of evolution (Figure 3) but, alternatively, it is hard to rule out that the presence of PolA in some phages is the result of a relatively late non-orthologous gene displacement.

It might not be possible to come up with decisive arguments rejecting one of the above scenarios, at least, at present. However, as already argued in some detail elsewhere [26], the primordial pool hypothesis (Figure 3) is simple, connects the origin of viruses and cells into one coherent scenario that is also linked to earlier stages of life's evolution, and seems to be best compatible with several lines of evidence. Perhaps, the most compelling of these is the existence of several "viral hallmark genes" that are shared by numerous, extremely diverse groups of viruses but are not found in any sequenced genomes of cellular life forms [26]. The primordial gene pool appears to be the natural source of the hallmark genes. Logically, the evolutionary succession of the DNA replication systems that is inferred here from comparative-genomic evidence also seems to be better compatible with this scenario for the early evolution of life. Indeed, under this scenario, the origin of the bacterial replication system is seen as evolution of a genetic element with a novel DNA polymerase that had limited success in an environment already inhabited by numerous elements with other, older replication systems, and gave rise to a single surviving line of descent, the bacteria. By contrast, the "proto-archaeal-LUCA" scenario (Figure 2) includes an additional, non-trivial step, the displacement of the ancestral replication with a new one in bacteria. This step appears to be all the less likely considering the paucity of PolC-based replication systems among modern viruses (Table 1) and their probable absence from ancient viruses: a virus to displace the archaeal-type replication system in the LUCA might not have been readily available. The primordial gene set scenario faces its own difficulties that, primarily, have to do with the conservation of several key membrane proteins in archaea and bacteria [54]. Ideas on the possibility of the evolution of such proteins in the context of intermediate stages of membrane evolution have been proposed [51,52,55] but remain to be developed into a coherent scheme. It should be noticed that, under this scenario, the emergence and diversification of PolB-base replication systems prior to the recruitment of PolC for the replicative function does not necessarily imply that proto-archaeal cells escaped from the networks of inorganic compartments earlier than proto-bacterial cells. Indeed, the capture of replication systems by emerging cells and their subsequent escape are likely to be uncoupled from the evolution of diverse replicons that might have reached considerable complexity during the pre-cellular stage of life's history.

The recent progress in comparative genomics of viruses has triggered a number of conceptual endeavors into the crucial links between origins and evolution of cells and viruses [23-26,56,57]. The (sometimes substantial) differences in the proposed evolutionary scenarios notwithstanding, these studies converge on the notion that "viruses take center stage in cellular evolution" [57]. The analysis presented here follows along the same lines by showing that a joint survey of viral and cellular genomes, in this particular case, for the presence of different enzymes of DNA replication, complemented by the comparative analysis of the respective protein folds, allows one to propose a provisional order for ancient evolutionary events (in this case, the origin of the archaeal-type and bacterial-type DNA replication systems) that otherwise appeared to be undecipherable. The hypothesis will be falsified if and when multiple and diverse groups of viruses are discovered that use bacterial PolC for their DNA replication; as discussed above, there is. presently, no indication of the existence of such a hidden continent of the virus world.

### Reviewers' comments

#### Reviewer's report 1

Eric Bapteste, Dalhousie University

Eugene V. Koonin's contributions to the field of evolutionary biology have been numerous although quite different in nature. This author (and his team) have provided rigorous scientific explanations for biological phenomena as well as contributed more prospective works, which point out important issues rather than propose robust answers. Such prospective works are important because they can indicate future directions for biological research. I consider the present manuscript to be of this second kind and to be meant to provide us with a hint of deeper evolution analyses still to come.

That is to say the reader should not consider the present manuscript as the last word on the question of the temporal order of evolution of the DNA replication systems, but as a good opportunity to think about it again. There could be and likely will be more to be said on this issue. If he wanted, and I think he could (thus maybe should), Eugene Koonin himself could contribute further and more decisive analyses on this topic in a slightly revised version of this manuscript.

Before I suggest some of the additional studies that could help test and maybe strengthen E. Koonin's current claim, I would like to stress an interesting perspective this paper could contribute to put forward. I take it to be the general and quite elegant idea, that "comparisons of viral genomes with the genomes of cellular life forms might provide windows into the deep past of life's evolution". This mine of genetic information is indeed not systematically explored in evolutionary analyses of cellular life forms, although, because it broadens the portion of the metagenome investigated, it would be certainly capable of highlighting the dynamics of cellular genome evolution. However, presenting a strong case for the use of the phylogenetic information stored in the DNA of viral communities remains challenging. In this regard, I am still unconvinced by the robustness of the claim of the present manuscript, although the temporal order of evolution of DNA replication systems presented here might be absolutely correct.

In this paper, Koonin presents a striking observation regarding the distribution of polymerases in viruses: the archaeal type (polB) is almost ubiquitous, the bacterial type (polC) is very rare. He thus legitimately looks for an explanation of this fact.

Several possibilities could a priori be considered:

(i) PolB is more broadly distributed because its fitness is better than polC fitness, and it invades viral genomes more efficiently: polB then replaces polC. One could not exclude that polB is a succesfull newcomer.

(ii) PolC is a newcomer in viral genomes, which was never able to successfully replace the efficient polB because polB is ancient and sucessful.

(iii) The present distribution does not really tell us much about which of these two polymerases appeared first, because we lack essential knowledge regarding the dynamic and mode of inheritance of polymerases within viral genomes. PolB and polC, in that regard, might well be equally ancient (and their present, highly unbalanced distribution could reflect the stochastic result of a very ancient competition between these two forms in the smallest first LUCAn population). Agnosticism is a scienfitic answer too.

In my view, a phylogeny of the viral polB to decide between these three options is currently lacking. Phylogenetic analysis of polB could test the presence of signs of high mobility and a tendency to spread across viral genomes. I would encourage Eugene Koonin to build such a phylogenetic tree of viral polB and to comment on it in a revised version of the manuscript.

**Author response: ***As noted by Bapteste himself, this is a discussion/hypothesis paper not a full-scale phylogenetic analysis. Given the huge number of sequences of polymerases and other replicative proteins that are currently available, the latter is a hard task. More importantly, the results are very difficult to interpret when viral and cellular sequences are mixed in the same tree because of the high and non-uniform rates of evolution that is typical of viruses and the inevitable long-branch problems associated with this pattern. These problems are quite apparent in recent phylogenetic analyses of diverse viral proteins including the B family polymerases. Surely, a variety of approaches can be used to cope with long-branch artifacts but that would be a different paper altogether. Of course, I cite the available phylogenies (refs*. [30,37]*) that, even as they suffer from the aforementioned problems, do provide some important indications, such as the apparent monophyly of PolB enzymes in the protein-primed replication systems*.

Furthermore, it would be interesting to test the congruence between the phylogeny of this marker and those of the other components of the replication systems. Congruence within the latter trees but disagreement with the former would suggest that polB has a tendency to be highly mobile, and capable of replacing native polymerases.

**Author response: ***Again, this suggestion certainly makes sense in principle; I am not at all denying the importance of phylogenetic analyses. There are, however, two reasons that compel me not to include any of these here. First, this is a large scale analysis that goes beyond the scope of this paper. Second, it is extremely hard to obtain reliable trees when viral sequences are analyzed jointly with homologs from cellular life forms. Information on phyletic patterns that is primarily used here, while limited in scope, is far more trustable*.

If this is not the case, I would be more inclined to accept Koonin's claim that maybe the poor distribution of polC reflects the relative recency of this polymerase (although I suspect the causes for such an extreme distribution could easily involve more than old historical causation alone). So far, I feel that the best argument presented in this manuscript in favor of polB ancestrality is not recorded in viruses DNA but rather in PolB structure. According to the author, PolB structure would be compatible with the RNA world hypothesis (and evolved from ancestral RdRp replicatives enzymes), while PolC structure would not be so, hence would be a secondary aquisition. I have no problem with this argument, except that then Eugene Koonin's point about the important role to be played by viral genomes content in ancient evolutionary analyses appear less important that I had hoped.

**Author response: ***I believe it is, exactly, the congruence of the two lines of argument that make the hypothesis on the primacy of PolB in the evolution of DNA replication worth a serious consideration. In the revised manuscript, I have modified the wording in the Abstract and in the Conclusions to emphasize this*.

The manuscript could also contain a more in depth biochemical presentation of PolB and PolC (structures, length, stability properties, etc.), which would help in the discussion of which polymerases has a higher chance of being carried/recruited by viral mosaic genomes, because some slight physico-chemical differences could not explain the predominance of one marker versus the other.

**Author response: ***I strongly believe there is no reason to think PolB is intrinsically "better" than PolC. Additional information and references on the catalytic efficiency of each enzyme family have been included in the revision*.

Finally, in all naivety, I am curious to know if some viral genomes investigated by Eugene Koonin happened to lack any of the currently known polymerases. If yes, does that mean that there are alternate replication systems, which could put the problem considered here in a different perspective by putting more attention on intermediary stages in the evolution of replication system, where neither polB nor polC were the decisive elements: after all in the egg-or-chicken-first controversy, the answer is a third term...Is it conceivable that there are even more polymerases (or proteins playing their role in an older replication system) to be discovered?

**Author response: ***Yes, quite a few viral genomes including some bacteriophages and archaeal viruses with reasonably large genomes and the eukaryotic polydnaviruses lack polymerases or other proteins involved in replication. In some cases (e.g., the classic phage λ), it has been directly demonstrated and, for other viruses in this category, it is assumed that the host replication machinery is recruited to replicate the viral genome. This was mentioned in the original manuscript but discussion of these "ultra-parasitic" viruses is expanded in the revision. I also argue why I do not believe in a "third path", i.e., the primacy of the ultimate dependence on the hosts in viral evolution, with subsequent acquisition of replicative genes, as the main route of viral evolution. In a nutshell, there are two reasons to prefer the scenario under which the first DNA viruses with large genomes encoded polymerases and other replicative proteins. First, viruses devoid of replicative proteins are relatively few and far between and, in the case of bacteriophages, are embedded within families that do encode their own replication machinery. Second, as extensively argued elsewhere (Ref*. [26]*), the wide spread of "viral hallmark genes", several of which encode replicative proteins, across all kinds of viruses stands as evidence of the prominence of idiosyncratic replication systems among ancient viruses. Admittedly, the viruses of hyperthermophilic crenarahaeota challenge both arguments, and this is also discussed in the revised version of the paper, in the special new section on Complications and Caveats*.

Minor questions:

- Could you please comment on the likely "age" of/relationships between the 8 phages genomes with a polC homologue? Does it look like polC was gained independently multiple times?

**Author response: ***Yes, this is addressed in the revision. Again and again, phylogeny of viral genes, in particular, an apparent lack of monophyly, should be treated with utmost caution. Nevertheless, at the face of it, it certainly looks like the polC gene might have been captured more than once*.

- All along the manuscript and in your figures, you refer to primitive life forms as bacteria or archaea. Do you really mean this? It would seem more correct to me to talk about cenancestor of bacteria/archaea (or populations of cenancestors of bacteria/archaea). To me bacteria is a label associated with extant lineages, but it is unclear wether we could identify such a bacteria in the past. When you use this term, do you have in mind a series of characters (bacterial synapomorphies) that made them a bona fide bacteria back then or are these bacteria and archaea of the past not that similar to our present ones (and maybe would deserve another name then)?

**Author response: ***A very curious and not so minor point that goes somewhat beyond the scope of the present manuscript. I have inserted "proto-archaea" and "proto-bacteria" here and there, to clarify the meaning. I could argue, however, that the actual reconstruction of the ancestral archaeal and bacterial genomes suggests that the last common ancestors of each of these domains of life, most likely, closely resembled relatively simple modern forms, i.e., they already were...archaea and bacteria. Perhaps, this is a subject to pick up on a future occasion*.

#### Reviewer's report 2

Patrick Forterre, Institut Pasteur

Eugene Koonin suggests in this paper to use information from the viral world to reconstruct the evolutionary history of the DNA replication apparatus. I completely agree with his conclusion that "*comparisons of viral genomes with the cellular life forms might provide windows into the deep past of lifes's evolution that appear to be out of reach for other approaches*". Here, Koonin specifically proposes that DNA polymerases of the B family (Pol B) originated before those of the C family (Pol C). His argument is that Pol B are much more widespread in different groups of organisms (cellular and viral) compared to Pol C (which is restricted to Bacteria and a few bacterial viruses). He suggests that "*the earlier diversification of viruses and virus-like entities using DNA Pol B occupied the major existing biological niches and thus prevented any significant diversification of selfish elements carrying Pol C*".

**Author response: ***Before proceeding with specifics, I must express my highest appreciation. Patrick Forterre's contribution to the study of DNA replication and its evolution and, more generally, to the field of early evolution of life and the evolutionary importance of viruses have been fundamental at many levels. I am honored and delighted to address these comments that, I believe, greatly add to the paper*.

The non-uniform distribution of the different DNA polymerases is indeed striking and requires an explanation. The merit of this paper is to focus for the first time on this point. However, the more general question is why Pol B are much more widespread in databases than DNA polymerases of any other families, since DNA Pol A, D, E, X and Y also exhibit a much more restricted distribution than DNA Pol B (Filée et al., 2002). If we apply the logic proposed by Koonin, the conclusion would be that Pol B originated first, followed by Pol A (universal in bacteria and widespread in their viruses, but also present in eukarya), Pol C, Pol D (only in euryarchaea) and Pol E (only in a few archaeal and bacterial plasmid). An enzyme such as the Topo V of *Methanopyrus kandleri *should be very recent since it has only been discovered in this archaeon (Forterre, 2006)! Such view would be quite simplistic and the real history has been certainly more complex.

**Author response: ***I have to state my disagreement. That very logic, simple as it might be, provides a reasonable ***null hypothesis ***on the succession of the evolutionary events, and moreover, when it comes to the most fundamental features of life, this might be the only way to come up with such a hypothesis. And, yes, the (relatively) recent origin of Topo V is an implication, and I do not think it is untenable. The resulting evolutionary scenario needs not to be simplistic inasmuch as it does not contradict the complexity brought about by horizontal gene transfer (which is the main point of Filée et al., 2002). Certainly, HGT events have been plentiful in evolution, including the evolution of DNA polymerases. The problem is not with HGT per se but with the possibility of HGT-driven sweeps obliterating the previous history and hence overturning the argument from diversity. Such sweeps are possible but I believe they should be pitted against the aforementioned null hypothesis, and the burden of proof is on those claiming a sweep (more on this below). I have included a brief discussion of this issue in the revised manuscript*.

The restricted distribution of a particular product of life evolution does not imply *per se *a more recent origin. For instance, when Neanderthals were still present in a corner of southern Europe, it would have been wrong (in the absence of fossil record) to conclude that *Homo sapiens sapiens *appeared first and occupied the major existing biological niches, thus prevented any significant diversification of more recent *Homo sapiens neanderthalis*! A protein with a restricted distribution thus could simply be an ancestral enzyme which has been later on displaced by a more successful functional analogue in the majority of lineages. It can be also a protein which was once widely distributed, but mainly in lineages without descendent today. The distribution of various proteins should be influenced by many factors, including their respective contribution to the fitness of the organisms in which they operate and the respective evolutionary success of the organisms bearing these proteins (two factors that can be either related or completely independent).

**Author response: ***There are several aspects of the human evolution analogy that are of interest. Under what circumstances would one arrive to the erroneous conclusion about the antiquity of H. sapiens and the late arrival of Homo sapiens neanderthalensis? I believe only if we had no knowledge of the spread of hominids outside Europe; no access to fossils; and no source of sequence or other genetic data. In other words, if our information was woefully incomplete. As soon as we got hold of any of these additional sources of information, we would see the distribution of humans in Europe for what it was, i.e., the H. sapiens sapiens population rapidly migrating to occupy most of Europe and the population heading toward extinction in the South. It should be noted that it is only a very superficial argument from diversity, namely, the diversity of geographical niches occupied by H. sapiens sapiens as opposed to H. sapiens neanderthalensis?, that would support the idea of Neanderthals being a recent offshoot of the human lineage. If there was a way to assess either the genetic diversity of the populations or the diversity of the fossil records, the fallacy of this hypothesis would quickly become apparent. Considering another, more fundamental issue in human evolution, the geographic origin of humans, the argument from diversity (of fossils and modern populations as well) has worked beautifully, being fully compatible with phylogeny and confidently pointing to the African cradle of humanity. Thus, to distinguish between "bad" and "good" arguments from diversity, additional evidence is important. Coming back to DNA polymerases, I think this is the exact situation with the hypothesis presented in the paper Firstly, the argument for the primacy of PolB is, truly, an argument from diversity (of genome layout and the organization of replication systems that center around PolB) not one from mere abundance. Secondly, this argument is congruent with a completely different line of evolutionary evidence, on based on the homology of PolB with other palm-domain polymerases*.

*Finally, I would like to point out a substantial difference between the evolution of species (like humans) and evolution of genes. Notwithstanding all the prominence of HGT in the latter, I do not believe it is possible to argue that it occurs as often and as readily as migration of organisms. This makes the analogy less pertinent and suggests that the argument from diversity might work better for genes than for species*.

*A brief discussion of the argument from diversity were added to the Complications and Caveats section*.

Coming back to DNA polymerases, the occurrence of Pol B and C in the three cellular domains cannot tell us much about their history, since we don't know the relative order of appearance of these domains (for instance, even if we were sure of the rooting of the universal tree in the "bacterial branch" the last common ancestor of bacteria might have existed either before or after the last common ancestors of the other two). We should then focus entirely on the viral world to try answering the question, why DNA Pol B are so abundant compared to others DNA polymerases ? This can be explained *a priori *by several non exclusive hypotheses beside the explanation proposed by Koonin:

1°) Pol B are more efficient than all the others, and thus many non-orthologous displacement occurred in which PolB displaced other DNA polymerases.

2°) there is a strong sampling bias in favour of viruses (plasmids) encoding Pol B in current databases

3°) the cellular hosts of viruses (plasmids) encoding Pol B have been preferentially selected in the game of evolution (for reasons having eventually nothing to do with their DNA polymerases).

I agree with Koonin that there is no reason to believe that Pol B are more efficient for processive DNA replication than Pol A or Pol C. Some Pol A are very efficient (for instance T7 DNA polymerase) and Pol C (at least in the presence of PCNA and other replication factors) can replicate bacterial chromosomes at an extremely high speed.

However, the two other explanations (bias sampling and/or disappearance of ancient hosts) should be seriously taken into consideration. Koonin argues against the idea of a sampling bias because "*the sequenced viral genomes already encompass a staggering diversity of gene repertoire and infect hosts from all main division of cellular life*". I quite disagree on this point. Most known bacterial viruses infect some groups of proteobacteria, some groups of Gram positive bacteria (firmicutes and mycobacteria) and cyanobacteria, i.e. 4 out of more than 30 bacterial divisions. More than half of the bacterial divisions detected by environmental PCR have no cultivable representative, thus no genome (cellular or viral) are available for these groups. Similarly, only a few archaeal lineages (mostly Sulfolobales and Halobacteriales) have been screened for viruses. In eukaryotes too, most superphyla have not been screened for the presence of viruses. We mainly know viruses of a few animals, plants and green algae.

A glimpse of the hidden virosphere can be obtained by looking at the ORFans in bacterial genomes. These ORFans are probably of viral or plasmid origin (Daubin and Ochman, Genome Res., 2004). However, in a recent analysis, Yin and Fisher (2006) found that only 2.8% of 110,186 bacterial ORFans have viral homologues. Their result suggests to me that we vastly underestimate the diversity of the modern virosphere. One therefore cannot exclude that viruses encoding Pol D or Topo V still exist, or that viruses encoding Pol C are abundant in bacterial, archeal or eukaryotic divisions which have not been studied.

**Author response: ***Of course, sampling biases are possible, moreover, inevitable, and no one can argue that a complete census of viruses existing in the biosphere is years ahead of us. Nevertheless, I think that the existing sampling is not negligibly small anymore and provides for detecting some credible trends, even as caution is always due. Is it likely that viruses in other bacterial divisions will be radically different from those in the four divisions that have been sampled? I think that likelihood is rather small, especially, given the interconnectedness of different "viromes", at least, in the sea (see references in the "virus world" paper; ref*. [26]).

*ORFans, I believe, provide a very biased glimpse of the virosphere, if any. Firstly, the viral/plasmid origin of ORFans, while an attractive hypothesis, is far from being supported by much hard evidence (see the paper of Yin and Fischer cited by Forterre). Secondly, more or less, by definition, ORFans are fast evolving proteins, so one would not expect to see many homologs in viruses unless the ORFan recently originated from a sequenced virus. I slightly expanded the discussion of potential effects of the sampling bias and added some extra words of caution in the revised manuscript*.

A recent metagenomic analysis by Rohwer and colleagues (Angly et al. 2006) even suggests that Pol C might be in fact even more widespread than Pol B in viruses infecting bacteria. Indeed, these authors found *polC *genes among the five most abundant enzyme-coding genes in three out of four oceanic viral metagenomes analyzed, whereas they never recovered genes encoding Pol B! Interestingly, they only recover genes encoding the alpha subunit of Pol C, suggesting that in viruses, this enzyme can be very processive alone. The existence of a large reservoir of polC genes in viruses supports the hypothesis that this bacterial replicase was initially recruited from a virus before the diversification of the bacterial domain (Forterre, 1999).

**Author response: ***The results of the metagenomic analysis of Rohwer and coworkers would present a very serious problem, perhaps, an actual falsification for the hypothesis that is put forward in this paper...if only these results could be taken at face value. However, a quick examination of Table 2 in Angly et al. shows that this is not the case. Indeed, in 3 out of the 4 explored metagenomic samples, PolC was among the most abundant "viral" enzymes. But what about the other enzymes that co-occur with PolC in those lists? These are a ribonucleotide reductase subunit, a formate dehydrogenase subunit, a carbamoyl-phosphate synthase subunit, a cytochrome oxidase subunit, 3-polyprenyl-4-hydroxybenzoate carboxylase, isoleucyl-tRNA synthetase, and methylcrotonyl-CoA carboxylase carboxyl transferase subunit. I deliberately listed those enzymes to convey the entire picture as one can glean it from the paper of Angly et al. Other than PolC, the list of the abundant enzymes includes only one found in some known viruses (not many), ribonucleotide reductase. The rest are typical bacterial proteins whose presence in viruses is both unprecedented and unlikely on biological considerations (very unlikely for some, e.g. cytochrome oxidase, an integral component of the membrane electron transfer chain). To me, examination of this list of abundant "viral enzymes" suggest an unequivocal, even if disturbing, even rather shocking conclusion: these enzymes are not viral but rather come from contaminating bacterial DNA. Thus, there is, at this point, still no defendable evidence of "a large reservoir of polC genes in viruses". Given the importance of this matter, I included a brief paragraph to this effect in the revised discussion section*.

Beside the sampling bias, one cannot exclude the historical bias. Viruses encoding Pol C polymerases might have existed for a very long time (predating or not those with PolB) but most of them might have disappeared with their hosts (except those infecting bacteria). The concept of lost lineages is presently neglected by Koonin and others evolutionists who base their argumentation on the principle of parsimony (see discussion with the reviewers in Koonin et al., 2006). Koonin put forward several time the argument of simplicity in favor of his hypotheses, he tell us that his general view of life history is *attractively simple*. I think that the path of history is precisely never so simple but usually extremely rich and complex. I would suggest that Koonin and others with similar views are using in fact an extreme form of "*actualism*", i.e. they want to explain all life evolution from its very beginning to the present state by only considering modern molecules and organisms (either cells or viruses). The combination of archaea and bacteria to produce eucaryotes is characteristic of this viewpoint. Incidentally, if Eukaryotes indeed originated from the association of a bacterium and an archaeon (as supported by Koonin) this paper would have no *raison d'être*, since all viruses and plasmids from eukaryotes encoding Pol B should be removed from Table 1 (being of archaeal origin). In that case (only two primary domains left), one cannot say that Pol B was prevalent in the ancient living world (before the origin of eukaryotes) since PolC is largely dominant in Bacteria (and probably in their viruses, considering the metagenomic previously discussed). The remaining question then would be, why Pol C (and Pol D) disappeared in Eukaryotes and their viruses?

**Author response: ***There are two very different, important issues brought up in this comment, the general (to some extent, philosophical) criticism of parsimony and actualism, and the relevance of eukaryotic viruses, and I address them separately. The point about parsimony, indeed, recapitulates the debate around the "virus world" paper but I find the issue to be so central to all historical sciences that it might be worth revisiting, even if briefly. The poverty of parsimony as a guiding principle in the reconstruction of history is obvious, and yet, I think the idea that "*the path of history is precisely never so simple but usually extremely rich and complex", *while not, exactly, wrong in itself, is going nowhere (or, worse, heading for disaster) as an epistemological principle. Yes, life is (staggeringly) complex but that does not mean that we should not proceed toward the reconstruction of its history by first constructing the simplest scenarios that are compatible with the available data and then rejecting them and replacing with more complex ones as soon as evidence of such extra complexity is obtained. I strongly believe that this is the only realistic path to progress in the study of evolution. "Occam all the way down", basically*.

*Regarding the problem of extinct lineages (this thread goes throughout Forterre's comments; I am trying to address it here all in one place). Of course, we are all well aware of the fact that most of the (eukaryotic) life forms that inhabited this planet throughout its history are not extant but extinct (I also like Gould's "Wonderful Life" quoted by Forterre below – of course, not at all the first recognition of the preponderance of extinct lineages but a beautiful expose). However, this is unequivocally true only for multicellular eukaryotes; we do not have a good idea at all just how many lineages of bacteria or archaea might have gone extinct. Of course, this is, largely, due to the near lack of fossil record although geochemical data might suggest the existence of such lineages. Actually, a combination of geochemistry and comparative genomic has recently led to the proposal of one such group, cyanobacteria-like organisms with anoxygenic photosynthesis *(Mulkidjanian et al. Proc Natl Acad Sci U S A. 2006;103:13126-31*). In general, however, the extent and role of extinction of major lineages in the evolution of prokaryotes is just not known*.

*Even in the case of eukaryotes, extinction is overwhelming only when relatively small twigs (species, genera, families) of the eukaryotic evolutionary tree are considered. For most of the evolutionary history of animals and plants, the spectrum of the phyla in existence remained the same. Apparently, during transition periods, like the Cambrian explosion, there have been considerable flourish of diverse animal phyla that, however, went extinct rather promptly. It stands to reason that early emergence of fundamentally diverse forms is, indeed, a general pattern in such transitional periods of life's evolution, including the origin of cells themselves. Most of these have been short-lived, so I see no reason to seriously doubt that, in the last 3 billion years or so, the only domains of life have been archaea and bacteria, and then, their derivative, the eukaryotes. In any case, Forterre's point is not just that there have been many extinct life forms but that their genetic heritage made substantial contributions to the genetic composition of the extant life forms. I think for this we need concrete evidence, and I maintain that such evidence is currently lacking. Specifically, with regard to eukaryotes, I just do not think there is any credible evidence of a contribution from a 3^rd ^domain; some more discussion of this issue can be found in another paper of mine recently published in Biology Direct (Koonin, Biol Direct. 2006;1:22) and a little more to follow below but a definitive study remains to be performed and described. The interesting and important issue of actualism is addressed in another comment below*.

*Here I must touch upon the issue of the relevance of eukaryotic viruses (those of them that have a DNA polymerase all encode PolB) to the raison d'être of the hypothesis presented in this paper. Indeed, at this point, I strongly support the concept of the origin of eukaryotes from an archaeal-bacterial symbiosis, without any contribution from a third, extinct domain (see more about this below). However, this does not automatically mean that the eukaryotic viruses that evolved in the wake of this symbiosis are entirely irrelevant for the problem of the temporal order of the origin of different replication systems. Considering that eukaryotic DNA viruses with large genomes definitely possess many genes of viral origin and likely evolved, primarily, via the sampling of the bacterial and archaeal viral pools (see Ref*. [26]*for discussion and references), the gene repertoire of eukaryotic viruses should reflect the gene composition of that ancient viral world. Under this scenario, the dominance of PolB among eukaryotic viruses seems to tell us that this was the primary choice of DNA polymerase available in the viral gene at the time of the emergence of eukaryotes, two billion years ago or so. I make it clear in the revised version of the manuscript that this is the context in which eukaryotic viruses are relevant for the present discussion*.

I don't want to argue here in detail with Koonin about the Martin and Russell hypothesis that life from the very origin to the emergence of Bacteria and Archaea originated and evolved in a single chimney. I would only mention that for me, this hypothesis is another example of *extreme actualism *(and simplification). *Actualism *means that you try to explain the past only with your knowledge about the present. This principle has been very powerful to fight the remnants of religious conceptions, especially in geology. However, the use of actualism in historical sciences is always delicate! In my opinion, the correct use of actualism in the present situation is to consider that known evolutionary patterns that occurred "recently" in the history of modern species also occurred much earlier in early life evolution. For example, we know that many lineages have been extinct during the evolution leading from the first animals to the modern fauna. Similarly, many *Homo *species have disappeared during the evolution leading from the ancestor of all *Homo *species to modern *Homo sapiens*. We can thus suppose, from the principle of actualism, that many cellular lineages also disappeared both during the evolution from the first cells to LUCA or from LUCA to the modern cellular world. In my opinion, an example of such lineages was probably the proto-eukaryotic lineages (urkaryote, *sensu *Woese), which were subsequently eliminated by modern eukaryotes harboring mitochondria (Kurland et al., 2006). Other lineages of ancient cells might have given birth to viruses in the RNA world (Forterre, 2006a). The existence of lost lineages would nicely explain the existence of the viral hallmark genes mentioned by Koonin, i.e. viral genes that are not present in modern cells.

This notion of lost lineages has been clearly depicted by Stephen Jay Gould in his book "*Wonderful Life*" in which he emphasized the notion of bottleneck, i.e. the fact that at several occasion in the course of evolution, massive extinctions occurred which specifically eliminate the majority of lineages present at that time on our planet. The emergence of *Homo sapiens *has produced such a bottleneck for the genus *Homo *by eliminating all other species that previously existed. As a consequence, all future *Homo *will be descendants of *H. sapiens*. Similarly, the emergence of LUCA should have produced a major bottleneck in the history of life, since all present cellular organisms originated from this ancestor. Three other bottlenecks probably also occurred at the origin of the three cellular domains.

**Author response: ***I agree on some basic points, namely, that, historically, actualism (and, more generally, uniformitarianism) has played an important and, to a large extent, positive role in affirming rational approaches to historical study, and also, that the application and implications of this principle are delicate. I also agree with Forterre's view of what is the appropriate use of actualism, i.e., trying to explain the past via causes now in operation (this is the principle that the same Stephen Jay Gould termed "methodological uniformitarianism" in a seminal early paper: Is uniformitarianism necessary? *American Journal of Science, 1965, 263: 223–28*). Somewhat ironically, however, should one apply uniformitarian thinking to the patterns of extinction, one would, of course, conclude that numerous lineages have gone extinct in the distant past, but should be very cautious, even skeptical about extinction of major taxa inasmuch as this is not commonly observable in the recent past (see above). A very different principle is what Gould called "substantive uniformitarianism": this is the notion, coming directly from Lyell, that not only the types of processes but also their rates and material conditions etc have been the same in the past as they are now. I will not say anything non-trivial by stating my belief that, while "methodological uniformitarianism" is correct and, basically, is the only reasonable way to do research in historical sciences, "substantive uniformitarianism" is, in the very least, overly restrictive and, effectively, wrong. However, I do not think I relied on substantive uniformitarianism as an assumption here or in previous papers. In particular, it has never been an assumption that extinct cell types did not contribute to the genetic composition of the modern cellular life forms and/or viruses. I cannot avoid using the "no extinct cellular lineages" as the null hypothesis but it is easy to imagine evidence that would falsify this hypothesis. In particular, if a 3^rd^, eukaryote-specific genetic system was decipherable from comparative-genomic data, I would readily entertain the idea of a third cellular lineage *sensu *Forterre. However, this is, demonstrably, not the case. Those genes that come across as eukaryote-specific (typically, with distant homologs in archaea or bacteria) belong to eukaryote-specific functions, such as cytoskeleton and cytoskeleton. This pattern is most economically explained by acceleration of evolution triggered by the functional shift, the latter, in turn, caused by the mitochondrial endosymbiosis*.

*To finish the discussion on actualism/uniformitarianism (although this is not the central subject of the present paper), I must submit that the non-cellular LUCA models actually represent a departure form substantive and, to some extent, even from methodological uniformitarianism. Indeed, these models picture the cenancestor of all modern cells as a non-cellular entity – an obvious departure from uniformitarian thinking, a more radical one, I believe, than ideas on displacement of membranes and membrane biogenesis pathways. I should also note that, when considering the earliest stages of life's evolution, in particular, the origin of translation and the genetic code, a break from any version of uniformitarianism is inevitable*.

*Thus, the status of actualism/uniformitarianism in evolutionary studies is, indeed, delicate and complex but I do not believe that the evolutionary scenarios discussed here rely on substantive uniformitarianism, not even implicitly. It is a completely different (and trivial) matter that we must relay on comparisons of extant genomes and organisms to make inferences about the past; we simply do not have other sources. The question is: is our data already sufficiently representative for the conclusions from comparative analyses to be credible at all. I try to argue here that, yes, that threshold has been crossed already (see above)*.

In summary, I think that we cannot presently really define the order of appearance of Pol B and Pol C because we have still not enough data to correctly estimate the sample bias. Furthermore, our answer to this question will remain speculative forever since we will never be able to fully reconstruct the history of lost lineages.

**Author response: ***It is hard to deny that an element of speculation will remain in inferences on very early evolutionary events, perhaps, "forever". Nevertheless, as already argued, I believe that the time is ripe to start seriously considering such scenarios, of course, not forgetting that corrections, perhaps, substantial ones will be required once we have more complete data*.

*It is my hope that the new section on Complications and Caveats makes this clear*.

The notion of lost lineages explains several puzzling observations, including the existence of hallmark viral genes of the combination of "orthologous" and non homologous proteins in the core of the DNA replication apparatus. Koonin and his colleagues explain this combination by the presence of DNA (but no DNA replication) in LUCA. Alternatively, I have suggested that the homologous DNA replication proteins present in the universal protein set were not present in LUCA but delivered by two or three different viruses at the onset of the three domains (Forterre, 2006b).

A final argument proposed by Koonin to suggest that Pol B appeared before Pol C is that the superfamily including Pol B includes the reverse transcriptase and cellular RNA polymerases (likely direct ancestor of cellular DNA polymerases) whereas the superfamily including Pol C includes nucleotidyl transferases (Bailey et al., 2006). However, the superfamily that includes Pol C also includes, beside Pol X and PolE, the PolyA polymerases (template independent RNA polymerase) and CCA adding enzymes (maturation of tRNA). The two superfamilies thus appear to be very old and were probably already diversified in the RNA world (with possibly some yet unknown RNA polymerases in both). Several DNA polymerases have probably originated independently in these two superfamilies from enzymes used in the RNA world.

**Author response: ***I do not much doubt that the enzymes of the polβ family have diversified already in the RNA world. The point is different, namely, that all these enzymes are non-replicative, typically, template-independent polymerases or nucleotidyltransferases, so it stands to reason that the origin of a DNA replication enzyme (PolC) from within this family is a late event*.

Although Koonin is still one of the rare evolutionists who fully recognizes the role played by viruses in early evolution (even suggesting that they originated before cells!), I have the feeling that he remains somewhat biased in this paper toward a cellular view of the world. Furthermore, this view is itself strongly biased toward his favourite scenario of cellular evolution. For instance, he divides the DNA replication mechanisms based on either Pol A or Pol C in two families, the "*Archaeal-type B family DNA polymerase *(either viral or cellular)" and the "*Bacterial-type C family DNA polymerase*" (especially in Table 1). In my opinion, these are not good expressions. The eukaryotic DNA replication mechanisms should not be labelled "Archaeal-type", since they include topoisomerases (Topo IB, Topo IIA) which have no orthologues in Archaea (Gadelle et al., 2003), several Pol B very distantly related to archaeal ones (Filée et al., 2002.) and several proteins involved in the initiation step which have no homologues in Archaea.

Why use the term Archaeal-type instead of eukaryotic type? This came clearly from a gradist view of evolution supported by Koonin and others in which eukaryotes derived from prokaryotes. Another consequence of the emphasis given to the procaryote/eukaryote dichotomy can be see in the expression "*prokaryotic and eukaryotic viruses*" used in the abstract). This formulation mixes archaeal and bacterial viruses (bacteriophages). As a consequence, archaeal viruses (grouped with bacteriophages) are presented under the headline "bacterial viruses" in the last edition of the viral taxonomy handbook (Fauquet et al., 2005). I suggest to Koonin and others to replace such expression by "viruses infecting archaea, bacteria and eukaryotes".

In the same vein, I found misleading to characterize a viral system by a cellular one and to talk about "*viruses replicating with the help of the archaeal system*". This reinforces the old conception in which viruses derived their proteins from modern cells. Also strange for me is the sentence "d*istinct complements of viruses were captured by escaping archaeal and bacterial cells"*. This formulation gives the active role to the cells, whereas it should be given to the viruses. Here is very helpful the very important advice of Jean-Michel Claverie to focus on the viral factory rather than on the virion, forcing us to consider that viruses are real living organisms which, beside cells, also have an active role in life evolution (Claverie, 2006).

**Author response: ***I edited the paper, modifying the wording in places, to avoid the impression that I stick to the old concept under which "viruses derived their proteins from modern cells"; obviously, I do not support this view (although some of them, like the NCLDV, indeed have derived a whole lot). However, I do not quite agree with the criticism of the phrases such as "*prokaryotic and eukaryotic viruses"*, "*viruses replicating with the help of the archaeal system*". Description of these replication systems by the name of the respective cellular domain is succinct and unequivocal, and does not at all imply "primacy" of cells in evolution (of course, Forterre and I fully agree that there is not such primacy – see Refs*. [24-26]*). Furthermore, I believe that the distinction between the viruses of prokaryotes (archaea and bacteria) and those of eukaryotes is meaningful inasmuch as the entirely real and substantial differences in cellular organizations between prokaryotes and eukaryotes affects many aspects of virus-cell interaction. Of course, this is an issue of biology (life style) not of taxonomy, and the position of the ICTV that lumps archaeal viruses with bacteriophages in the current taxonomic scheme is disingenuous. Finally, I should note that the notion of the derivation of eukaryotes from prokaryotes (via endosymbiosis) has nothing to do with gradism or any other pre-conceived "ism". From my point of view, it is, simply, the most economical explanation for the origin of the eukaryotic cell we can think of today. Of course, one can be swayed by philosophical pre-conceptions unwittingly and subconsciously but I strongly doubt it is the case for this particular conundrum*.

Finally, I have one historical remark. In the Background section, lane 6. Koonin writes that "*it came as an extraordinary surprise when comparative genomics ushered in the realization that the protein components of the DNA replication systems are not universally conserved*".

Interestingly, this was in fact predicted by Carl Woese and George Fox in 1977 in their very important paper on "*The concept of cellular evolution*". These two authors quote that "*certain enzymes involved in DNA replication should appear quite dissimilar in the two cases *(eukaryotes and bacteria) because they predicted that their common ancestor (the progenote) was still a member of the RNA world. The big divide between the two systems became apparent as soon as the genes encoding *E. coli *DNA Pol III and eukaryotic DNA Pol α, δ or ε became available (see Forterre et al., 1994). However, it is true is that the problem remained largely ignored until the Mushegian, Koonin's PNAS paper of 1996.

**Author response: ***I am pleased to restore the historical precision. The text has been modified accordingly, and both Woese and Fox (1977) and Forterre et al. (1994) are now cited*.

#### Reviewer's report 2: reference list

Angly, F.E., et al. The marine viromes of four oceanic region, PLoS Biol., 4 (2006)

Bailey, S., Wing, R.A. and Steitz, T. The structure of T. aquaticus DNA polymerase III is distinct from eukaryotic replicative DNA polymerases. Cell.126:893 (2006)

Claverie, J.M. Viruses take center stage in cellular evolution. Genome Biology, 7:110 (2006).

Daubin, V. and Ochman, H. Start-up entities in the origin of new genes. Curr Opin Genet Dev 14:616 (2004)

Fauquet, C. et al. Virus Taxonomy. VIII edition, San Diego and London, Elsevier, Academic Press (2005)

Filée, J., Forterre, P., Sen-Li, T. and Laurent, J. Evolution of DNA polymerase families: evidences for multiple gene exchange between cellular and viral proteins. J. Mol. Evol., 54, 763 (2002)

Forterre, P. DNA topoisomerase V: a new fold of mysterious origin. Trends in Biotechnology, 24, 245 (2006)

Forterre, P. Displacement of cellular proteins by functional analogues from plasmids or viruses could explain puzzling phylogenies of many DNA informational proteins Mol. Microbiol. 33, 457 (1999)

Forterre, P. The origin of viruses and their possible roles in major evolutionary transitions Virus Res. 117(1):5–16 (2006a)

Forterre, P. Three RNA cells for ribosomal lineages and three DNA viruses to replicate their genomes: a hypothesis for the origin of cellular domain Proc. Natl Acad Sci U S A. 103:3669 (2006b)

Forterre, P., et al., M. Evolution of DNA topoisomerases and DNA polymerases : A perspective from Archaea. System. Appl. Microbiol. 16, 746 (1994).

Gadelle, D., Filée, J., Bukher, C. and Forterre, P. Phylogenomics of Type II DNA topoisomerases Bioassays. 25: 232 (2003)

Koonin EV, Senkevich TG, Dolja VV.The ancient Virus World and evolution of cells. Biol Direct. 1:29 (2006)

Kurland CG, Collins LJ, Penny D.Genomics and the irreducible nature of eukaryote cells. Science. 312:1011 (2006)

Mushegian, A.R. Koonin, E.V. A minimal gene set for cellular life derived by comparison of complete bacterial genomes. Proc. Natl. Acad. Sci. 93:10268 (1996)

Woese, C.R. and Fox G. The concept of cellular evolution. J Mol Evol. 10:1 (1977)

Yin, Y. and Fischer, D. On the origin of microbial ORFans: quantifying the strength of the evidence for viral lateral transfer. BMC Evol Biol. 16: 63 (2006).

#### Reviewer's report 3

Mark Ragan, University of Queensland

Referee comments on Koonin, Temporal order of evolution of distinct DNA replication systems inferred by comparison of cellular and viral DNA polymerases

This paper outlines a scenario for the emergence of DNA replication systems. The scenario is built primarily on the distribution of different DNA polymerases among organisms and viruses, but gains support from mechanistic and protein-fold data. The argument contributes to the current tendency to bring viruses closer to "centre stage in cellular evolution".

My only significant concern – not only about this paper – is that this line of argument relies significantly on the assumption that present-day viruses are representative of viral lineages that extend back to the beginnings of cellular evolution. Of course we routinely make such assumptions about cellular organisms: early archaea almost certainly had the same archaeal-type PolB polymerases, distinctive membrane lipids and so on that are uniformly distributed among today's archaea. Even so, there is compelling evidence that individual genes have been acquired, duplicated, broken up, shuffled about and lost over time. But as viruses are much simpler, and particularly as some are agents of genetic transfer, with what confidence can we infer unbroken lineages of viral descent, and unchanged contents of viral genomes, over several billion years? Sampling issues aside, could not the wide distribution PolB in viruses be due, at least in part, to more-recent origins and/or selective sweeps?

**Author response: ***These are, certainly, pertinent considerations but I think I will not include any comments here because the issue has been addressed in considerable detail in the recent paper on the evolution of the "Virus World" (Ref*. [26]).

## Competing interests

The author declares that he has no competing interests.

## Authors' contributions

EVK conceived of the hypothesis, performed the computational analyses, and wrote the article.
